# Association among blood pressure, antihypertensive drugs, and amyotrophic lateral sclerosis

**DOI:** 10.1055/s-0045-1804922

**Published:** 2025-05-13

**Authors:** Zhiguang Li, Yan Li, Jiankai Zhao, Feifei Zhang, Wei Dang, Yanan Jia, Fei Guo, Lixin Guo

**Affiliations:** 1 Xingtai Central Hospital, Department of Neurology, Xingtai Hebei, People's Republic of China.; 2 Xingtai Medical College, Department of Basic Medicine, Xingtai Hebei, People's Republic of China.; 3 Xingtai Central Hospital, Department of Science and Education, Xingtai Hebei, People's Republic of China.; 4 Xingtai Central Hospital, Department of Cardiac Surgery, Xingtai Hebei, People's Republic of China.

**Keywords:** Blood Pressure, Antihypertensive Agents, Mendelian Randomization Analysis, Amyotrophic Lateral Sclerosis

## Abstract

**Background** Amyotrophic lateral sclerosis (ALS) is a fatal and incurable neurodegenerative disease. The impacts of antihypertensive drugs and blood pressure (BP) on ALS are currently debatable.

**Objective** To evaluate the causal relationship involving antihypertensive drugs, BP, and ALS through a Mendelian randomization (MR) analysis.

**Methods** The causal relationship between BP and ALS was evaluated by a bidirectional two-sample MR analysis. Then, a sensitivity analysis was performed using a secondary BP genome-wide association study. The drug-target MR was employed to evaluate the impact of antihypertensive drugs on ALS. Furthermore, we used cis-expression quantitative trait loci (cis-eQTLs) data from brain tissue and blood to validate the positive results by a summary-based MR method.

**Results** We found that an increment in systolic BP (SBP) could elevate the risk of ALS (inverse-variance weighted [IVW] odds ratio [OR] = 1.003; 95% confidence interval [95%CI]: 1.001–1.006; per 10-mmHg increment) and ALS might be protected by angiotensin-converting enzyme inhibitors (ACEIs; OR = 0.970; 95%CI: 0.956–0.984; *p* = 1.96 × 10 ^−5^ ; per 10-mmHg decrement). A causal relationship was not observed between diastolic BP and other antihypertensive drugs in ALS.

**Conclusion** In the present study, genetic support for elevated SBP serves as a risk factor for ALS. Besides, ACEIs hold promise as a candidate for ALS.

## INTRODUCTION

Amyotrophic lateral sclerosis (ALS) is a fatal and incurable neurodegenerative disease. Its incidence escalates as age advances, with a pronounced peak occurring between ages 60 and 79. 1 The incidence of hypertension rises as age progresses and high systolic blood pressure (SBP) emerges as the predominant risk factor for global disability-adjusted life-years and all-cause mortality. 2 Hypertension is not uncommon in ALS patients. Therefore, evaluating the causal relationship between antihypertensive medications (AHMs), blood pressure (BP), and ALS contributes to clinical decision making.

According to recent observational studies, ALS patients may present some comorbidities before disease onset, with hypertension being the most common. 3 Notwithstanding, the impact of blood pressure on this disease is still uncertain. Previous studies revealed hypertension is a risk for ALS, 4 5 while others displayed hypertension as a mitigating factor. 6 7 The same applies to the correlation between AHMs and ALS. The risk of ALST may be down-regulated by the treatment with angiotensin-converting enzyme inhibitors (ACEIs), as illustrated by a population-based case-control study. 8 Additionally, various subsequent observational studies and meta-analyses found that AHMs could lower the incidence of ALS. 9 10 11 However, there were also trials that had not reached reliable positive conclusions. 12 The inconsistencies in the above findings were largely attributed to the influence of reverse causality, sample size limitations, and confounding factors. In the absence of interventions to prevent or cure the disease, experimental strategies targeting drug repurposing have come to the hotspot of research, offering promising therapeutic potential for ALS.

Mendelian randomization (MR) is a new genetic statistical method. The causal relationship between outcome and exposure was assessed by MR utilizing genetic variations. Besides, it can address certain weaknesses found in conventional observational studies in predicting the association of risk factors by modelling randomized controlled trials (RCTs) with naturally clustered risk alleles. 13 14 Moreover, drug-target MR has the ability to deliver essential information about medicines. Variations within or adjacent to drug target genes can influence their expression, and the impact of drugs on disease can be predicted by the genetic effects. 15 To offer a theoretical basis for disease prevention as well as clinical medication decision-making, the correlation between BP and ALS was investigated by a bidirectional two-sample MR. Subsequently, a drug-target MR was employed to evaluate the impact of five classes of first-line AHMs on ALS: ACEIs, beta-blockers (BBs), thiazide diuretic agents, calcium channel blockers (CCBs), and angiotensin receptor blockers (ARBs). 16

## METHODS

### Study design

**Figure 1:**
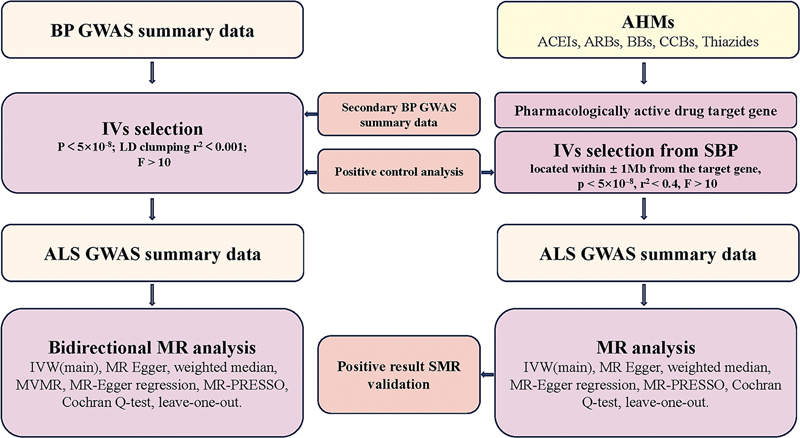
Abbreviations: ACEIs, angiotensin-converting enzyme inhibitors; AHMs, antihypertensive medications; ALS, amyotrophic lateral sclerosis; ARBs, angiotensin receptor blockers; BBs, beta-blockers; BP, blood pressure; CCBs, calcium channel blockers; GWAS, genome-wide association study; IVW, inverse-variance weighted; LD, linkage disequilibrium; MR, Mendelian randomization; MVMR, multivariable MR;SBP, systolic blood pressure; SMR, summary-based MR. Schematic representation of the study design.

First, the causal effect between ALS and BP traits was explored using a bidirectional two-sample MR. To avoid the interactions between DBP and SBP, we utilized a multivariable MR (MVMR) method to access the impact of BP traits on ALS. Secondly, the causal effects of AHMs on ALS were examined by drug-target MR. Additionally, positive control analysis was performed and the summary-based MR (SMR) method was used to validate the results. The reporting was consistent with the recommendations outlined in the Strengthening the Reporting of Observational Studies in Epidemiology Using Mendelian Randomization (STROBE-MR) statement. 17 Figure 1 displayed the overview of the study design.

### Data for BP and AHMs

Single nucleotide polymorphisms (SNPs) were extracted from the largest genome-wide association study (GWAS) of SBP and diastolic blood pressure (DBP), which involved 757,601 individuals of Europeans descent from the UK Biobank and the International Consortium of Blood Pressure database, with adjustments for sex, age, and body mass index (BMI). 18 Detailed information was presented in the **Supplementary Material 1** ( https://www.arquivosdeneuropsiquiatria.org/wp-content/uploads/2024/11/ANP-2024.0077-Supplementary-Material-1.xlsx ), **Table S1** (online only). Detailed information on the summarized data sources for the instrumental variables is presented in **Table S2** (online only).

The BP was assessed by manual or automated measurement, and defined as a continuous numerical variable. The mean DBP of the study was 84.3 mmHg (standard deviation [SD] = ± 11.3), adjusted for AHMs use by adding of 10 mmHg. The mean SBP of the study was 141.1 mmHg (SD = ± 20.7), adjusted for the use of AHMs by adding 15 mmHg. Additionally, secondary GWAS summary data of BP offered by the UK Biobank served as a sensitivity analysis to illustrate the robust findings. Notably, such data did not adjust for BMI. 19 The selection of instrumental variables (IVs) was strictly restricted to those that were independently (r ^2^ < 0.001) linked to BP at the genome-wide significance (GWS) level ( *p* < 5 × 10 ^−8^ ).

The regulatory or encoding regions of pharmacologically active targets of AHMs was identified by the DrugBank and GeneCards online platforms ( **Tables S3** , **S4** , online only). The selection of IVs for each AHM was mainly based on the methods previously published by Gill et al, 20 and we also referred to a study by Luo et al. 21 Simply put, IVs were characterized by SBP-related genetic variants in relatively modest linkage disequilibrium (LD, r ^2^ < 0.4) and at a GWS level ( *p* < 5.0 × 10 ^−8^ ). Notably, the range of genetic variants was only within 1 megabase upstream and downstream of target gene ( **Table S5** , online only). To check the robustness of the positive results, with reference to Chauquet's method, 22 the SMR method was employed to perform a sensitivity analysis on statistically significant antihypertensive drug target genes. This method utilized the expression quantitative trait loci (eQTLs) of drug target gene as exposure to analyze the impact of genetic variances of drug target gene on ALS. In this study, genetic instruments were only generated by two sets of cis-eQTLs from blood and brain tissue, used in SMR analysis. The summary-level data obtained for cis-eQTLs were from the study by Qi et al. 23 ( https://yanglab.westlake.edu.cn/pub_data.html ) and the eQTLGen Consortium( https://www.eqtlgen.org/ ).

### Data for positive control analysis

We validated the IVs selection for BP and AHMs through positive control analysis. Due to the widely recognized protective role of AHMs in coronary heart disease (CHD), we examined the association of IVs with this disease. The summary data of CHD was from CARDIoGRAMplusC4D Consortium.

### Data for outcome

The ALS GWAS summary statistics were sourced from the Project MinE consortium, the largest available ALS dataset to date, comprising 110,881 controls of European ancestry and 27,205 ALS cases. 24 To date, the compilation of GWAS data on ALS is the largest. In reversed MR study, the selection of IVs for ALS were based on independence (r ^2^ < 0.001) and at GWS level ( *p* < 5.0 × 10 ^−8^ ).

### Statistical analysis

According to the Mendelian randomization principle, selected genetic variances need to meet three assumptions:

- An association between the exposure of interest and the selected IVs;
- The selected IVs are unaffected by potential confounders;
- The IVs that were selected only influence the findings via the exposure of interest and not via other pathways.

The overall causality between outcome and exposure was determined by the random-effect inverse-variance weighted (IVW) method as a primary analysis method. The robust outcome of IVW was measured using the MR Egger method and the weighted median (WM) method as sensitivity analysis. The MR Egger regression and MR Pleiotropy RESidual Sum and Outlier (MR-PRESSO) were adopted to analyze the potential horizontal pleiotropy of the IVs. A *p* -value < 0.05 (Cochran Q-test) was indicative of evidence of heterogeneity.

The leave-one-out analysis was performed to determine the likelihood of whether a single SNP was able to drive the outcome or not. Presence of linkage in the observed association in SMR method was measured by the heterogeneity in dependent instruments (HEIDI) test. The use of F-statistics could evaluate whether outcomes were susceptible to weak instrument bias. 25 The SNPs with an F-statistic > 10 were only included to mitigate the deviations resulting from weak IVs. The causal impact of DBP and SBP on ALS risk was calculated by scaling to a 10 mmHg increase at BP levels. On the contrary, the association of AHMs on ALS was measured by a 10 mmHg reduction in SBP, which represented the therapeutic effect across various classes of AHMs.

The 95% confidence interval (95%CI) with corresponding odds ratio (OR) exhibited the outcomes. The causal association represented the statistical significance threshold following Bonferroni correction for AHMs ( *p* < 0.01 [0.05/5]) and BP ( *p* < 0.013 [0.05/4]). A suggestive association was found in *p* -value greater than 0.013/0.01 but lower than 0.05. This present study was performed by the Two-Sample MR (open source, version 0.5.6) in the statistical program R (R Foundation for Statistical Computing, Vienna, Austria), version 4.2.2, and the SMR software (Yang Lab, Philadelphia, PA, United States), version 1.03.

## RESULTS

### Positive control analysis

**Figure 2:**
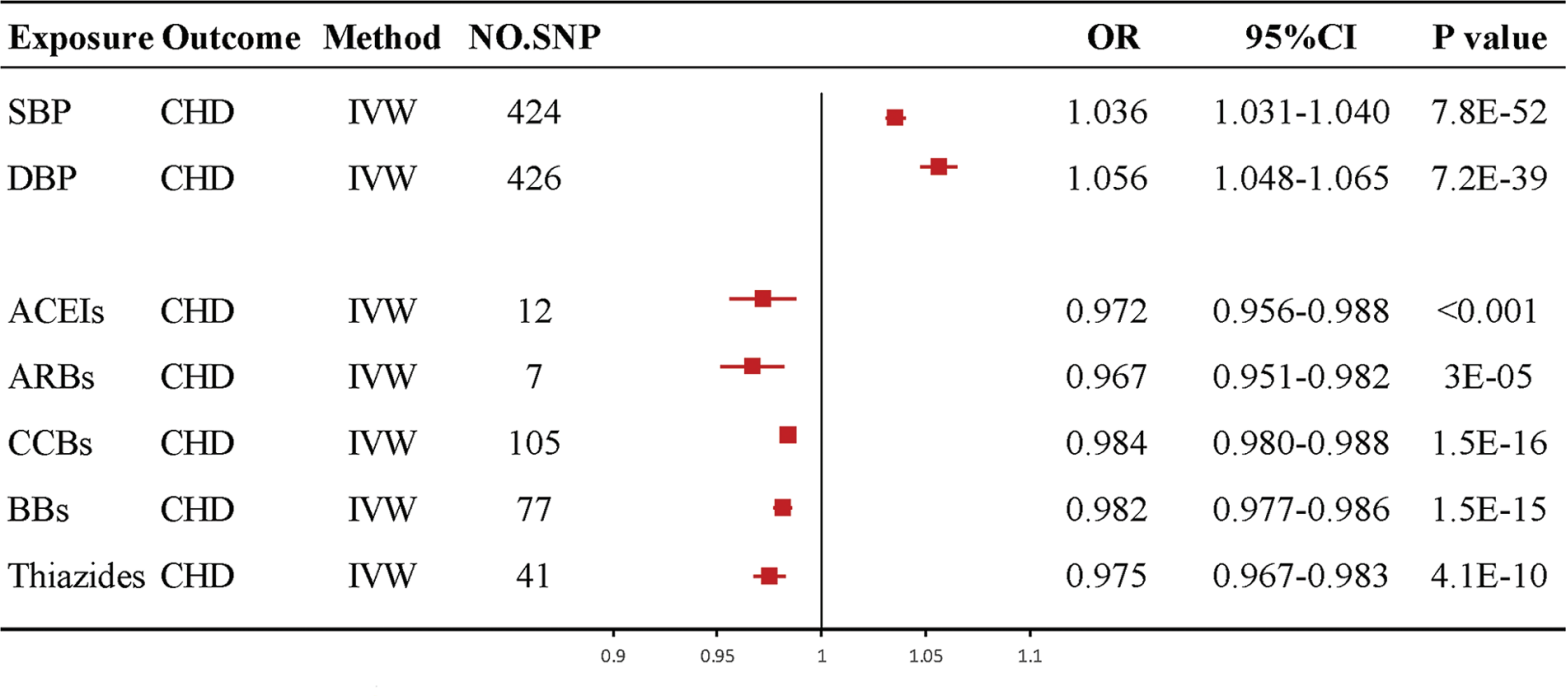
Abbreviations: 95%CI, 95% confidence interval; ACEIs, angiotensin-converting enzyme inhibitors; AHMs, antihypertensive medications; ARBs, angiotensin receptor blockers; BBs, beta-blockers; BP, blood pressure; CCBs, calcium channel blockers; CHD, coronary heart disease; DBP, diastolic blood pressure; IVW, inverse-variance weighted; MR, Mendelian randomization; No.SNP, number of SNPs; OR, odds ratio; SBP, systolic blood pressure; SNP, single nucleotide polymorphism. The MR analyses involving genetically-predicted BP, AHMs, and CHD risk by IVW method. The OR and 95%CIs were scaled to each 10-mmHg increment for BP traits, and 10-mmHg lower in SBP for AHMs.

The positive control analysis confirmed that genetically predicted SBP and DBP were significantly associated with an increased risk of CHD ( *p* < 0.05; per 10-mmHg increment), as shown in Figure 2 . Additionally, ACEI, ARBs, BBs, and CCBs all reduced the risk of CHD ( *p* < 0.05; per 10-mmHg decrement), except for thiazide diuretic agents ( Figure 2 ). In a word, the findings of positive control analyses confirmed the accuracy of the IVs selected in this study.

### The association between BP and ALS

**Figure 3:**
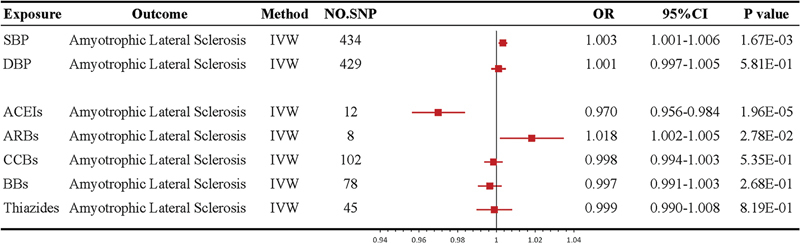
Abbreviations: 95%CI, 95% confidence interval; ACEIs, angiotensin-converting enzyme inhibitors; AHMs, antihypertensive medications; ALS, amyotrophic lateral sclerosis; ARBs, angiotensin receptor blockers; BBs, beta-blockers; BP, blood pressure; CCBs, calcium channel blockers; DBP, diastolic blood pressure; IVW, inverse-variance weighted; MR, Mendelian randomization; No.SNP, number of SNPs; OR, odds ratio; SBP, systolic blood pressure; SNP, single nucleotide polymorphism. The MR analyses between genetically predicted BP, AHMs, and ALS risk by IVW method. Both OR and 95% CIs were scaled to each 10 mmHg increment for BP traits, and 10-mmHg lower in SBP for AHMs.

Our MR analysis revealed that genetically elevated SBP is associated with an increased risk of ALS (IVW OR = 1.003; 95%CI: 1.001–1.006; per 10-mmHg increment, Figure 3 ). The robust results of the IVW analysis were validated by directional consistency of WM and MR Egger analysis results ( **Table S6A** , online only). The MR PRESSO global test was applied to observe the horizontal pleiotropy for IVs ( *p* < 10 ^−4^ ), but the outcome of the MR Egger intercept did not illustrate the existence of horizontal pleiotropy (intercept = 0.001; *p* = 0.756) ( **Table S7** , online only).

The heterogeneity for IVs was analyzed on the grounds of Cochran's Q test (Q = 540.105; *p* = 0.0003) ( **Table S7** , online only). Notwithstanding, MR results were not invalidated, for we choose random-effect IVW. The removal of any one of the IVs would not change the results of the leave-one-out analysis. Visualization results were exhibited in the Supplementary Material 2 and Figure S1A (online only).

When adjusting DBP, MVMR showed this causal relationship still existed (OR = 1.006; 95%CI: 1.001–1.010; *p* = 0.016; per 10-mm Hg increment). When we used secondary GWAS statistics of BP provided by the UK Biobank, the above findings regarding the effect of BP on ALS were supported by IVW method, WM method and MR Egger method simultaneously ( **Table S6C** , online only). The visualized IVW, WM, MR Egger and Leave-one-out results were shown in Figure S1B , online only.

A causal correlation was not found between DBP and ALS (IVW OR = 1.001; *p* = 0.581; Figure 3 ; MR Egger OR =1.006; *p* = 0.199; WM OR = 1.002; *p* = 0.518), as shown in **Table S6A** . The evidence of heterogeneity for MR estimates was identified by Cochran Q test, but the results would not be affected. Based on the MR PRESSO global test, the horizontal pleiotropy for IVs was suggestive. However, such results were not corroborated by the MR Egger intercept ( **Table S7** , online only). After the adjustment of SBP, MVMR revealed a robust result of DBP on ALS (OR = 0.995; 95%CI: 0.987–1.002; *p* = 0.167; per 10-mm Hg increment). Upon the use of secondary GWAS statistics on DBP offered by the UK Biobank, the causal effect of DBP on ALS was not represented in the outcome ( **Table S6C** , online only). The visualized IVW, WM, MR Egger, and leave-one-out results were displayed in Figure S2A , **B** (online only).

No evidence of ALS-induced changes in BP trait was found in reverse MR. There was no heterogeneity and horizontal pleiotropy ( **Tables S6B** and **S7** , online only). The visualized IVW, WM, MR Egger and Leave-one-out results were shown in Figures S3 S4 (online only).

**Figure 4:**
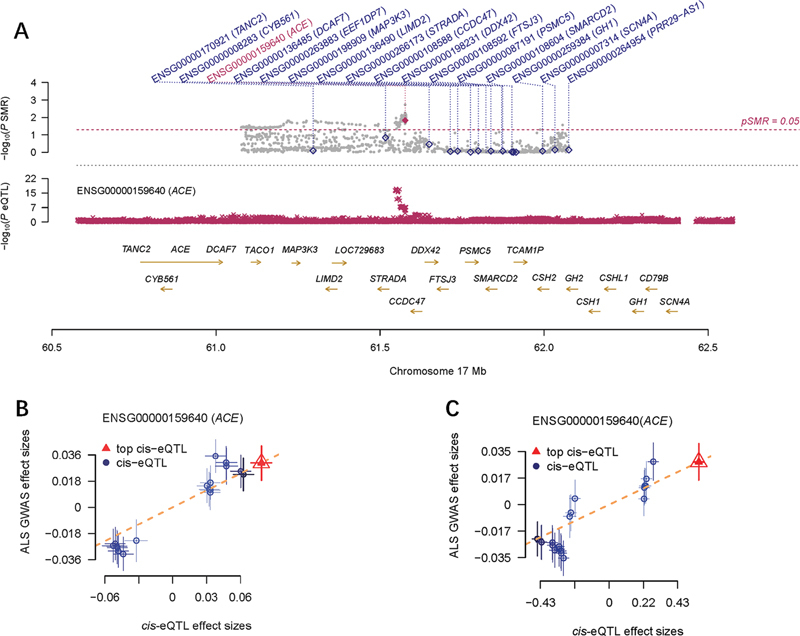
Abbreviations: 95%CI, 95% confidence interval; ALS, amyotrophic lateral sclerosis; cis-eQTLs, cis-expression quantitative trait loci; GWAS, genome-wide association study; MR, Mendelian randomization; OR, odds ratio; SMR, summary-based MR. The MR analyses between *ACE* gene expression and ALS risk by SMR method. A LocusZoom plot showing the consistent genetic effects of ALS GWAS and cis-eQTLs nearby *ACE* ( *p* < 0.05). The B SMR analysis between *ACE* gene expression in blood and ALS GWAS. The C SMR analysis between *ACE* gene expression in brain and ALS GWAS.

### Antihypertensive drug effect on ALS risk

We found that genetic proxies for ACEIs protected against ALS (IVW OR = 0.970; 95%CI: 0.956–0.984; *p* = 1.96 × 10 ^−5^ ; Figure 3 ; WM OR = 0.970; 95%CI: 0.952–0.988; *p* = 0.001; per 10-mmHg decrement), as shown in **Table S8** , (online only). A MR Egger analysis revealed the consistency with the direction of IVW analysis (OR = 0.963; 95%CI: 0.894–1.038; *p* = 0.349; per 10-mmHg decrement). A relationship was observed between suggestive higher risk for ALS and genetically predicted ARBs (OR = 1.018; 95%CI: 1.002–1.005; *p* = 0.028; per 10-mmHg decrement), as shown in **Table S8** (online only). No horizontal pleiotropy or heterogeneity was revealed in the MR analysis of ACEIs and ARBs on ALS ( **Table S9** , online only). For BBs, CCBs, and thiazides, no causal association with ALS was observed ( **Table S9** , online only). The visualized IVW, WM, MR Egger, and leave-one-out results were shown in **Figures S5** , **S9** (online only).

We further validated the effect of ACEIs, ARBs and ALS using the SMR method. First, we used cis-eQTLs from eQTLGen Consortium to access the role of ACEIs and ARBs on ALS. Besides, we found that ACEIs has a protective effect against ALS (SMR OR = 0.847; *p* = 0.015; per SD decrement; Figure 4 ), as shown in **Table S10** (online only), was consistent with IVW analysis based on the encoding-region method. But the causal relationship was not displayed between ARBs and ALS ( **Table S10** , online only). Second, *ACE* gene was expressed in brain. Based on this, we used cis-eQTLs from brain tissue to perform a secondary SMR analysis. The protective effect of ACEIs had also been demonstrated (SMR OR= 0.979; *p* = 0.021; per SD decrement; Figure 4 ), as shown in **Table S10** (online only). According to the HEIDI test, the linkage did not cause the observed associations ( *p* > 0.05).

## DISCUSSION

This study suggested that the effect of BP was explored by a two-sample MR research and we commonly prescribed AHMs on ALS using the largest GWAS scale for BP and ALS. Genetically determined SBP was found to increase the risk of ALS and ACEIs were beneficial for ALS, which may have significant implications for ALS prevention and clinical treatment decisions. Although the IVW analysis showed that ARBs had a suggestive risk for ALS, this had not been validated by the SMR analysis.

Hypertension is a well-known and common disease in the middle-aged and elderly population. It has detrimental effects on multiple organs and systems throughout the body, leading to damage in several target organs such as the kidneys, brain, heart etc. The effect of BP on ALS is currently not fully elucidated and some observational studies have shown inconsistent results, as mentioned above.

However, RCTs on ALS are difficult to conduct for ethical considerations and it is difficult to address the shortcomings of traditional observational studies. Hence, the causal relationship between blood pressure and ALS was predicted by an MR analysis. Primary method suggested increased SBP were able to elevate the risk of ALS in this study. When we used secondary BP GWAS data for our exposure, we came to a consistent conclusion. After adjusting for DBP, the results were still significant. The destructive effect of SBP on ALS may be due to insufficient cerebral perfusion caused by hypertension, which may accelerate neuronal degeneration. 26 27 Notwithstanding, the specific mechanism by which blood pressure affects ALS is not yet clear and the hypothesis requires more evidence.

The renin-angiotensin system (RAS) is a well-recognized endocrine system. This system plays a significant physiological role in modulating body fluid volume as well as the cardiovascular system of the peripheral circulation. Additionally, RAS also acts in the central nervous system and affects brain function and disorders. This system is linked to the pathogenesis and modulation of Alzheimer's disease. 28 The angiotensin type 1 receptor (AT-1R) axis/ACE/angiotensin II (Ang II) is implicated in increasing the activation of cell apoptosis, neuroinflammation and oxidative stress. 29

The angiotensin converting enzyme (ACE) serves as an essential component of RAS. Several studies have explored how ACE inhibitors may reduce neuroinflammation and oxidative stress, both key factors in ALS pathology. 30 31 These mechanisms suggest that ACE inhibitors may provide neuroprotective effects beyond their role in blood pressure regulation, although further mechanistic studies are required to confirm this hypothesis in the context of ALS. In drug-target MR, we found that ALS may be protected by genetically determined ACEIs. The effect of ACEIs on ALS was supported by SMR method using cis-eQTLs summary data in both blood and brain. Furthermore, our findings regarding the protective effect of ACEIs on ALS were consistent with a previous case-control study. 8 There are multiple biological effects of ACEIs, and their protective effect in ALS may not be due to their direct antihypertensive effect.

In the brain RAS, angiotensinogen is mainly expressed as astrocytes in various regions of the brain, then converted into several peptides to play physiological roles. 28 Among them, ACE 1 converts angiotensin I to II (AT-I to -II), which can increase the activation of cell apoptosis, neuroinflammation, and oxidative stress. 29 Also, ACE contributes to the pathogenesis of neurodegenerative diseases. 32 33 The main effect of ACEIs could selectively inhibit ACE 1 and reduce the production of AT-II.

Additionally, ACEIs may protect against ALS through different mechanisms. One of the neuropathological signatures of ALS is the activation of astrocytes and microglia. 30 Some studies demonstrated that the activation of the hippocampal microglia and astrocyte can be suppressed by perindopril, 31 34 and can improve the resulting brain dysfunction. It should be noted that ACEIs can extend the half-life of bradykinin, thereby influencing the glial inflammatory response and reducing its impact; 35 they could also scavenge the hydroxyl radical, 36 reduce glutamate-induced neurotoxicity, 37 and restore the level of α-tocopherol, 38 which could protect against ALS. More clinical evidence and mechanism research is needed to confirm this.

Our study used the largest ALS GWAS database currently available and obtained consistent conclusions using different BP GWAS data as exposure. In the drug target MR, we used summary data from different tissue sources to mutually verify the robustness of the conclusion. Notwithstanding, our interpretation of the results should still be more cautious.

There are still some limitations in our study. First, while MR provides an opportunity to explore genetic associations, it cannot definitively establish causality. Our findings suggest potential causal relationships between blood pressure traits and ALS; however, these results reflect associations rather than direct causal effects. The inherent limitations of MR, including the possibility of residual confounding and pleiotropy, prevent us from making definitive causal claims. Second, unmeasured confounders may still influence the results, despite the use of statistical methods such as MR-Egger and MR-PRESSO to address pleiotropy. Factors such as lifestyle or environmental influences, which were not accounted for, may have biased our results. Third, our findings are primarily based on GWAS data from European ancestry populations, limiting generalizability to other ethnic groups. Further research, involving more ethnically diverse populations, is necessary to validate these associations across different ancestral groups and ensure broader applicability. Finally, without effective stratified GWAS data, it is not possible to assess the impact on patients with different forms of the disease and different rates of progression.

In conclusion, our study suggested that the genetic support for elevated SBP as a risk factor for ALS and ACEIs holds promises as a potential candidate for ALS.

## References

[1] Feldman E L Goutman S A Petri S Amyotrophic lateral sclerosis Lancet 2022 400(10360):1363 138010.1016/S0140-6736(22)01272-7 36116464 10.1016/S0140-6736(22)01272-7 PMC10089700

[2] GBD 2017 Risk Factor Collaborators Collaborators G BDRF Global, regional, and national comparative risk assessment of 84 behavioural, environmental and occupational, and metabolic risks or clusters of risks for 195 countries and territories, 1990-2017: a systematic analysis for the Global Burden of Disease Study 2017 Lancet 2018 392(10159):1923 199410.1016/S0140-6736(18)32225-6 30496105 10.1016/S0140-6736(18)32225-6 PMC6227755

[3] CARE-MND Consortium Glasmacher S A Kearns P KA Larraz J Prevalence of multimorbidity and its impact on survival in people with motor neuron disease Eur J Neurol 2021 28 08 2756 276510.1111/ene.14940 34036680 10.1111/ene.14940

[4] Xu K Ji H Hu N Cardiovascular comorbidities in amyotrophic lateral sclerosis: A systematic review J Clin Neurosci 2022 96 43 4910.1016/j.jocn.2021.12.021 34974247 10.1016/j.jocn.2021.12.021

[5] Moreau C Brunaud-Danel V Dallongeville J Modifying effect of arterial hypertension on amyotrophic lateral sclerosis Amyotroph Lateral Scler 2012 13 02 194 20110.3109/17482968.2011.610110 21913867 10.3109/17482968.2011.610110

[6] Hollinger S K Okosun I S Mitchell C S Antecedent disease and amyotrophic lateral sclerosis: What is protecting whom? Front Neurol 2016 7 4710.3389/fneur.2016.00047 27065942 10.3389/fneur.2016.00047 PMC4810157

[7] Lian L Liu M Cui L Environmental risk factors and amyotrophic lateral sclerosis (ALS): A case-control study of ALS in China J Clin Neurosci 2019 66 12 1810.1016/j.jocn.2019.05.036 31155341 10.1016/j.jocn.2019.05.036

[8] Lin F C Tsai C P Kuang-Wu Lee J Wu M T Tzu-Chi Lee C Angiotensin-converting enzyme inhibitors and amyotrophic lateral sclerosis risk: a total population-based case-control study JAMA Neurol 2015 72 01 40 4810.1001/jamaneurol.2014.3367 25383557 10.1001/jamaneurol.2014.3367

[9] Abdel Magid H S Topol B McGuire V Hinman J A Kasarskis E J Nelson L M Cardiovascular diseases, medications, and ALS: a population-based case-control study Neuroepidemiology 2022 56 06 423 43210.1159/000526982 36481735 10.1159/000526982 PMC9986836

[10] Pfeiffer R M Mayer B Kuncl R W Identifying potential targets for prevention and treatment of amyotrophic lateral sclerosis based on a screen of medicare prescription drugs Amyotroph Lateral Scler Frontotemporal Degener 2020 21(3-4):235 24510.1080/21678421.2019.1682613 31684770 10.1080/21678421.2019.1682613 PMC9930913

[11] Hu N Ji H Medications on hypertension, hyperlipidemia, diabetes, and risk of amyotrophic lateral sclerosis: a systematic review and meta-analysis Neurol Sci 2022 43 09 5189 519910.1007/s10072-022-06131-7 35616813 10.1007/s10072-022-06131-7

[12] Franchi C Bianchi E Pupillo E Angiotensin-converting enzyme inhibitors and motor neuron disease: An unconfirmed association Amyotroph Lateral Scler Frontotemporal Degener 2016 17(5-6):385 38810.3109/21678421.2016.1143515 26913547 10.3109/21678421.2016.1143515

[13] Davies N M Holmes M V Davey Smith G Reading Mendelian randomisation studies: a guide, glossary, and checklist for clinicians BMJ 2018 362 k60110.1136/bmj.k601 30002074 10.1136/bmj.k601 PMC6041728

[14] Haycock P C Burgess S Wade K H Bowden J Relton C Davey Smith G Best (but oft-forgotten) practices: the design, analysis, and interpretation of Mendelian randomization studies Am J Clin Nutr 2016 103 04 965 97810.3945/ajcn.115.118216 26961927 10.3945/ajcn.115.118216 PMC4807699

[15] Schmidt A F Finan C Gordillo-Marañón M Genetic drug target validation using Mendelian randomisation Nat Commun 2020 11 01 325510.1038/s41467-020-16969-0 32591531 10.1038/s41467-020-16969-0 PMC7320010

[16] Wright J M Musini V M Gill R First-line drugs for hypertension Cochrane Database Syst Rev 2018 4 04 CD00184110.1002/14651858.CD001841.pub3 29667175 10.1002/14651858.CD001841.pub3 PMC6513559

[17] Skrivankova V W Richmond R C Woolf B AR Strengthening the Reporting of Observational Studies in Epidemiology Using Mendelian Randomization: The STROBE-MR Statement JAMA 2021 326 16 1614 162110.1001/jama.2021.18236 34698778 10.1001/jama.2021.18236

[18] Million Veteran Program Evangelou E Warren H R Mosen-Ansorena D Genetic analysis of over 1 million people identifies 535 new loci associated with blood pressure traits Nat Genet 2018 50 10 1412 142510.1038/s41588-018-0205-x 30224653 10.1038/s41588-018-0205-x PMC6284793

[19] L EB . MRC IEU UK Biobank GWAS pipeline version 12017

[20] Gill D Georgakis M K Koskeridis F Use of genetic variants related to antihypertensive drugs to inform on efficacy and side effects Circulation 2019 140 04 270 27910.1161/CIRCULATIONAHA.118.038814 31234639 10.1161/CIRCULATIONAHA.118.038814 PMC6687408

[21] Luo S Schooling C M Wong I CK Au Yeung S L Evaluating the impact of AMPK activation, a target of metformin, on risk of cardiovascular diseases and cancer in the UK Biobank: a Mendelian randomisation study Diabetologia 2020 63 11 2349 235810.1007/s00125-020-05243-z 32748028 10.1007/s00125-020-05243-z

[22] Chauquet S Zhu Z O'Donovan M C Walters J TR Wray N R Shah S Association of antihypertensive drug target genes with psychiatric disorders: A Mendelian randomization study JAMA Psychiatry 2021 78 06 623 63110.1001/jamapsychiatry.2021.0005 33688928 10.1001/jamapsychiatry.2021.0005 PMC7948097

[23] Qi T Wu Y Fang H Genetic control of RNA splicing and its distinct role in complex trait variation Nat Genet 2022 54 09 1355 136310.1038/s41588-022-01154-4 35982161 10.1038/s41588-022-01154-4 PMC9470536

[24] SLALOM Consortium PARALS Consortium SLAGEN Consortium SLAP Consortium van Rheenen W van der Spek R AA Bakker M K Common and rare variant association analyses in amyotrophic lateral sclerosis identify 15 risk loci with distinct genetic architectures and neuron-specific biology Nat Genet 2021 53 12 1636 164810.1038/s41588-021-00973-1 34873335 10.1038/s41588-021-00973-1 PMC8648564

[25] Bowden J Del Greco M F Minelli C Davey Smith G Sheehan N A Thompson J R Assessing the suitability of summary data for two-sample Mendelian randomization analyses using MR-Egger regression: the role of the I2 statistic Int J Epidemiol 2016 45 06 1961 197410.1093/ije/dyw220 27616674 10.1093/ije/dyw220 PMC5446088

[26] Mandrioli J Ferri L Fasano A Cardiovascular diseases may play a negative role in the prognosis of amyotrophic lateral sclerosis Eur J Neurol 2018 25 06 861 86810.1111/ene.13620 29512869 10.1111/ene.13620

[27] Daulatzai M A Cerebral hypoperfusion and glucose hypometabolism: Key pathophysiological modulators promote neurodegeneration, cognitive impairment, and Alzheimer's disease J Neurosci Res 2017 95 04 943 97210.1002/jnr.23777 27350397 10.1002/jnr.23777

[28] Gouveia F Camins A Ettcheto M Targeting brain Renin-Angiotensin System for the prevention and treatment of Alzheimer's disease: Past, present and future Ageing Res Rev 2022 77 10161210.1016/j.arr.2022.101612 35346852 10.1016/j.arr.2022.101612

[29] Abiodun O A Ola M S Role of brain renin angiotensin system in neurodegeneration: An update Saudi J Biol Sci 2020 27 03 905 91210.1016/j.sjbs.2020.01.026 32127770 10.1016/j.sjbs.2020.01.026 PMC7042626

[30] Masrori P Van Damme P Amyotrophic lateral sclerosis: a clinical review Eur J Neurol 2020 27 10 1918 192910.1111/ene.14393 32526057 10.1111/ene.14393 PMC7540334

[31] Dong Y F Kataoka K Tokutomi Y Perindopril, a centrally active angiotensin-converting enzyme inhibitor, prevents cognitive impairment in mouse models of Alzheimer's disease FASEB J 2011 25 09 2911 292010.1096/fj.11-182873 21593435 10.1096/fj.11-182873

[32] Alzheimer Disease Genetics Consortium (ADGC) European Alzheimer's Disease Initiative (EADI) Cohorts for Heart and Aging Research in Genomic Epidemiology Consortium (CHARGE) Genetic and Environmental Risk in AD/Defining Genetic, Polygenic and Environmental Risk for Alzheimer's Disease Consortium (GERAD/PERADES) Kunkle B W Grenier-Boley B Sims R Genetic meta-analysis of diagnosed Alzheimer's disease identifies new risk loci and implicates Aβ, tau, immunity and lipid processing Nat Genet 2019 51 03 414 43010.1038/s41588-019-0358-2 30820047 10.1038/s41588-019-0358-2 PMC6463297

[33] International FTD-Genomics Consortium Ge Y J Ou Y N Deng Y T Prioritization of drug targets for neurodegenerative diseases by integrating genetic and proteomic data from brain and blood Biol Psychiatry 2023 93 09 770 77910.1016/j.biopsych.2022.11.002 36759259 10.1016/j.biopsych.2022.11.002 PMC12272336

[34] Bhat S A Goel R Shukla R Hanif K Angiotensin receptor blockade modulates NFκB and STAT3 signaling and inhibits glial activation and neuroinflammation better than angiotensin-converting enzyme inhibition Mol Neurobiol 2016 53 10 6950 696710.1007/s12035-015-9584-5 26666666 10.1007/s12035-015-9584-5

[35] Asraf K Torika N Apte R N Fleisher-Berkovich SMicroglial Activation Is Modulated by Captopril: in Vitro and in Vivo Studies. Front Cell Neurosci.2018;12:116. Epub 20180501. doi: 10.3389/fncel.2018.00116. PubMed PMID: 29765306; PubMed Central PMCID: PMCPMC5938337. 10.3389/fncel.2018.00116 PMC5938337 29765306

[36] Ravati A Junker V Kouklei M Ahlemeyer B Culmsee C Krieglstein J Enalapril and moexipril protect from free radical-induced neuronal damage in vitro and reduce ischemic brain injury in mice and rats Eur J Pharmacol 1999 373 01 21 3310.1016/s0014-2999(99)00211-3 10408248 10.1016/s0014-2999(99)00211-3

[37] Sengul G Coskun S Cakir M Coban M K Saruhan F Hacimuftuoglu A Neuroprotective effect of ACE inhibitors in glutamate - induced neurotoxicity: rat neuron culture study Turk Neurosurg 2011 21 03 367 37110.5137/1019-5149.JTN.4313-11.0 21845573 10.5137/1019-5149.JTN.4313-11.0

[38] Michal Freedman D Kuncl R W Weinstein S J Malila N Virtamo J Albanes D Vitamin E serum levels and controlled supplementation and risk of amyotrophic lateral sclerosis Amyotroph Lateral Scler Frontotemporal Degener 2013 14 04 246 25110.3109/21678421.2012.745570 23286756 10.3109/21678421.2012.745570 PMC3673294

